# Factors associated with provision of physical activity in primary schools in Makindye Division in Kampala, Uganda: a cross-sectional study

**DOI:** 10.1186/s12889-023-15216-7

**Published:** 2023-02-11

**Authors:** Lena Mpalampa, Stephen Okoboi, Sarah Maria Nabaggala, Rose Clarke Nanyonga

**Affiliations:** 1 Lifelink Hospital, P.O. Box 3074, Kampala, Uganda; 2 Deputy Head of Research Department, Infectious Diseases Institute, Makerere University, P. O. Box 22418, Kampala, Uganda; 3 Infectious Diseases Institute, Makerere University, P. O. Box 22418, Kampala, Uganda; 4 Clarke International University, P.O. Box 7782, Kampala, Uganda

**Keywords:** Physical activity, Primary school, Physical education, Sufficient physical activity, Insufficient physical activity, School children

## Abstract

**Background:**

Globally, school-going children spend most of their days at school, sitting in lessons and unable to achieve the daily WHO recommendations for Physical Activity (PA) of at least 60 minutes per day. Limited studies have assessed the opportunities schools provide for PA to help the children achieve their daily recommended PA. We determined the level of and the factors associated with PA offered in primary schools in Makindye Division in Kampala during the school term.

**Methods:**

This cross-sectional study was conducted in 36 selected government and private primary schools in Makindye Division, Kampala. PA was defined as the amount of time in minutes available for PA as per WHO recommendations – sufficient (60 minutes or more of PA) or insufficient (less than 60 minutes of PA) and assessed for any factors associated with provision of time for PA in schools. Data were collected by interview administered questionnaires and analysed descriptively. Factors associated with PA were assessed using a logistic regression model.

**Results:**

Of the 36 schools, 3 were government and 33 were private schools. The proportion of schools offering sufficient time for PA among Primary schools in the Makindye Division was (8/36) 22%. The average time for PA for all schools per week was 197 minutes (SD 70.7). Sufficient PA was associated with the provision of PE lessons after a break or after lunch (*p*-value 0.038). Sufficient PA was more likely in schools that offered volleyball (OR 8.69), had space in the school for PA (OR 13.27), provided athletics (OR 2.26) and whose fees were Ushs 700,000 (USD 187) or more (OR 1.30).

**Conclusions:**

Only 22% of sampled schools offered sufficient time for PA among Primary schools in Kampala per WHO guidelines. Provision of sufficient time for PA was associated with PE scheduled either after break or lunch. Sufficient PA was more likely with schools that had space for PA, schools which offered volleyball and athletics, and whose fees were 700,000/= or more.

Schools should consider scheduling PE lessons after break or after lunch to increase the likelihood of meeting the targeted time for PA. Primary schools need to be supported to establish facilities and to increase diversity in available activities to ensure children achieve their recommended PA.

## Background

Globally, an alarmingly high percentage (81%) of adolescents aged 11-17 years are insufficiently active [1], with very few children around the world (27 -33%) able to achieve their daily recommended PA target [2]; and these figures have been largely unchanged over the past few years. Specifically, in the UK, only 22% of 5-15-year-olds met the target, while in the USA, only 23.2% of children 14-18 years were sufficiently active [3]. Across Africa, the figures vary from only 12.6% in Nairobi, Kenya [4] to 25% in Ghana [5] and 36.3% in Kampala, Uganda [6].

The average duration offered for PA by primary schools was only 103 minutes per week [7] way below the recommended sufficient target of at least 60 minutes of PA daily [8]. Furthermore, only half of the school children seem to take part in school activities with much lower numbers being reported in low-income countries, where less than a third of children participate in school activities [2].

Physical activity (PA) in children is essential because it contributes to the healthy growth and development of musculoskeletal tissues, coordination, and socio-psychological wellbeing [9]. These benefits track into adulthood reducing the risk for premature all-cause mortality, development of non-communicable diseases (NCDs), and improving mental health well-being [10–12]. Today, however, children spend much of their time in school seated during lessons or later doing homework. As such, very little time is left for children to participate in physical activities (PA) outside school [13, 14]; yet children aged 5-17 years should accumulate, on average, 60 minutes of moderate - to vigorous-intensity physical activity daily, including muscle and bone strengthening [8, 9].

Most of the studies on PA in children worldwide, including those in Uganda, focus on an individual child’s activity. However, as insufficient PA in children continues to increase worldwide [15] contexts, such as schools, in which school-going children spend the majority of their time, need to be assessed to see if they are providing adequate opportunities for PA to help children achieve their daily recommended PA. The school setting is therefore ideal for promoting PA because there is easy access to a large number of children at the same time, and the PA interventions can easily emphasize good health practices which can be sustained [16, 17].

Emphasis on PA in schools stems from the World Health Organization (WHO) target to reduce the global prevalence of physical inactivity in adolescents and adults by 15% by 2030; as such, guidelines and policies have been written for countries to either adopt or develop their national policies or action plans to help them meet the target [18]. However, despite the presence of international guidelines and targets [8], as well as the Ministry of Education and Sports' (MoES) promotion of physical education in Uganda [19], insufficient PA in children persists.

Limited studies have assessed the opportunities schools provide for PA to help the children achieve their daily recommended PA. We, therefore, determined the level of and the factors associated with PA offered in primary schools in Makindye Division in Kampala during the school term.

## Methods

### Aim

Since most studies focus on the individual child’s activity, there is a paucity of data in Uganda on a) how much time is offered for physical activity in primary schools to enable children to achieve the recommended national and international guidelines; and b) any factors which may be hindering the provision of sufficient PA in schools. Therefore, this study assessed the physical activities offered in primary schools in Makindye Division in Kampala and the factors influencing these practices.

### Study design

The study used a cross-sectional analytical design to describe factors that influence PA provision and the time offered for PA in primary schools in Kampala. Data was collected during the COVID-19 pandemic when schools were temporarily opened to allow a few classes to return to school. Since contact sport was not permitted, school representatives interviewed at each school had to recall some routine PA conducted in schools before the pandemic. The study was conducted over 1 month in February of 2021.

### Study setting

The study was carried out in the Makindye division in Kampala, the Capital City of Uganda. Makindye is one of the divisions of Kampala District which is made of 5 divisions. It has 83 Primary Schools: 6 are government schools which represents 7.2% of the 83 licensed schools, and 77 are private schools representing 92.8% of the 83 schools [20].

### Characteristics of study population

The study population was any Licensed Primary schools in Makindye Division, Kampala which met the inclusion criteria: having all the 7 classes (primary 1 through 7), being open during the study period and those who gave consent to participate in the study.

#### School selection

Identification and selection of schools according to criteria were carried out by the Principal Investigator (PI). For the schools which met the selection criteria, a schedule was made for the research assistant or PI to visit the schools.

The head teacher was approached for consent by the PI or research assistant. The schools whose head teachers gave consent were then recruited and assigned a study number. The questionnaire was then administered by the PI or research assistant to either the head teacher, administrator, or the physical education teacher. No personal identifying factors were obtained.

### Sample size

To calculate the sample size, we used the formula for the sample size for a finite population. Where 4% is the proportion of schools that provided sufficient physical activity daily for children in elementary schools in the USA [21]. This generated a sample size of 36.

Stratified sampling was used. The schools were divided into 2 strata: government and private schools and then proportionate sampling was done. From our calculated sample size of 36, 7.2% (3) were government schools and 92.8% (33) were private schools. Simple random sampling was used to select participants from each stratum.

### Outcome variables

The primary outcome of this study was the amount of time offered for physical activity in primary schools as reported by a school representative; and it was categorised based on – whether it was sufficient or insufficient. Sufficient PA was measured as 60 minutes or more of daily PA, while insufficient PA was less than 60 minutes of daily PA as per WHO guidelines for each child. The principle of accumulation was followed to calculate the total minutes scheduled for each week [8].

#### Covariates

We included covariates such as School Ownership, sex of the attending students, type of school (day, boarding, or mixed), and religious foundation (affiliation) of the school.

Teacher-related factors included the number of teachers available, and the qualifications of the PE teachers.

Class-related factors included: the number of students in the class and the age of the students.

School-related factors included: the presence of a timetable for PA, availability of space for PA, availability of equipment for PA, availability of curriculum for PA, time when PE is scheduled, availability of optional additional PA activities after school, and type of PA offered.

### Data collection

Data were collected through interview administered questionnaire to the school representative in the sampled schools. The interviewee was someone answering on behalf of the school - either the Head teacher, Administrator, or the Physical Education teacher. The tool was a pre-coded, pretested structured questionnaire with mainly closed-ended questions.

### Statistical analysis

Data were cleaned, coded, and double-entered into Epidata version 3.1 software packages and imported into STATA version 13.0 for data cleaning and analysis [22].

#### Data analysis

A descriptive analysis was done using the univariate analysis to describe the baseline characteristics of the sampled schools. The distribution of the school characteristics was presented as frequencies with respective proportions for categorical parameters while means and standard deviations were presented for continuous parameters. The primary study outcome, sufficient PA (60 minutes or more of PA daily) or insufficient PA (less than 60 minutes of PA daily) was presented as a proportion. Different types of physical activities were presented as categorical variables. These include active play during the break, ball games like football, netball or athletics, and gymnastics.

Variables such as school fees and the number of students in the class were presented as means (standard deviation) if normally distributed or as medians (inter quintile range) if not normally distributed.

Bivariate descriptive analysis was done to determine relationships between variables and PA. Multivariate analysis was done to measure if the associations between the factors and PA persisted when controlled for confounders.

Factors assessed included the presence of trained PE teachers, curriculum, set time on timetable, facilities, and equipment for PA among others. All variables with a relatively small *p*-value (*p* < 0.5) at the bivariate stage were investigated further at the multivariate stage using a logistic regression unless otherwise indicated. The variables that showed statistical significance in the bivariate analysis were included in the multivariate analysis to determine the factors independently associated with adherence to MOE and WHO PE time guidelines i.e., with the provision of sufficient PA.

The binary logistic regression model determined the existence and strength of the association between guidelines and the independent variables. A binary logistic regression was used due to the dichotomous nature of the dependent variable (adherence to the guidelines); where one meant adherence (Sufficient PA) and 2 meant non-adherence (insufficient PA).

The multivariate logistic regression adopted in the status of adherence was based on the formulae:

1 $$\ln \left[\frac{{\textrm{p}}_{\textrm{i}}}{\textrm{l}-{\textrm{p}}_{\textrm{i}}}\right]={\beta}_0+{\beta}_1{\textrm{X}}_1+\dots +{\beta}_k{\textrm{X}}_{\textrm{k}}$$

Where;

X_i_ = independent variables

p_i_ = Probability of adhering to guideline

l − p_i_ = Probability of not adhering to guidelines

*β*_0_ = constant

*βi*= coefficient of the determinant

Logistic regression used the log odds ratio and presented an association in form of an odds ratio with a corresponding confidence interval of 95%. OR > 1 meant the independent variable had a significant association with adherence to the guidelines i.e., Sufficient PA, OR < 1 showed that the independent variable did not have an impact on school adherence to MOE and WHO guidelines i.e., insufficient PA. OR = 1 showed no association between the independent variable and sufficient PA.

## Results

Of the 83 primary schools in Makindye Division, 36 schools met the eligibility criteria and were included in the data analysis. 33 (91.7%) were private schools and 3 (8.3%) were government schools. All 36 schools were mixed-gender schools. 18 (50%) were day schools only, 18 (50%) were mixed (day and boarding). The baseline characteristics of the schools are summarised in Table 1.

**Table 1:** Baseline characteristics of the schools

Characteristics	Level	Number (36)	Percentage (% = 100)
Type of school	Day	18	50.0
	Boarding	0	0.0
	Mixed	18	50.0
Sex of students	Single-sex school	0	0.0
	Mixed-sex school	36	100.0
School ownership	Public	3	8.3
	Private	33	91.7
Religious foundation	Pentecostal	9	25.0
	None	10	27.8
	Other: COU, Catholic, Islam	17	47.2
School fees	< 700,000 Ugx	26	72.2
	≥700,000 Ugx	10	27.8
	Mean (SD)	512,768.1 (417,833.7)	

### Time for physical activity

The number of primary schools in Makindye Division which provided sufficient time for PA according to the WHO guidelines (> 60 min per day) were only 8 (22.2%).

The average time for PA offered by all schools per week before the COVID 19 pandemic was 197 minutes (SD 70.7) with the average time for PA for lower classes (P.1 - P.3) with younger children at 196.8 (SD 75.34), P.4 - P.6 was 195.11(71.79) and P.7 was 196.13 (SD 72.41).

### Physical activity during break time

All the schools provided breaks during the day. In most schools, 28 (77.7%) provided 30 minutes for morning break, while 4 (11.11%) provided 20 minutes, and 3 (8.33%) provided 45 minutes. The average time for the break was 29.86 (SD 4.54). In all schools, half the break time was used for snacks and half the time was available for the students to engage in active play.

### Types of physical activities offered during PE and after-school activities

The types of PA the children were involved in during the scheduled PE time and the after-school activities were categorised into those using balls, doing athletics, and gymnastics.

In the ball category, 34 (94%) schools met the Uganda MoE&S guidelines of offering at least 2 items. All 36 schools offered football. For the athletics category, 19 (52.8%) schools offered at least 2 options of activities while 16 (44.4%) schools did not offer any activity in the gymnastics category. Swimming was offered in only 4 (11.1%) schools, all of which were private (See Table 2).

**Table 2:** Summary of the types of physical activities offered by schools

Type of physical activity	Frequency Offered	Number (36)	Percentage (% = 100)
Activities offered in the ball category	Not Offered	0	0
	1	2	6
	≥2	34	94
Football	Not offered	0	0.0
	Offered	36	100.0
Netball	Not offered	4	11.1
	Offered	32	88.9
Volleyball	Not offered	21	58.3
	Offered	15	41.7
Activities offered for athletics	None	13	36.1
	1	4	11.1
	≥ 2	19	52.8
Javelins	Not offered	23	63.9
	Offered	13	36.1
Short puts	Not offered	24	66.7
	Offered	12	33.3
Discus	Not offered	24	66.7
	Offered	12	33.3
Activities offered in gymnastics	Not Offered	16	44.4
	Offered	20	55.6
Swimming lessons	Not offered	32	88.9
	Offered	4	11.1

### Teacher and class related factors

The average size of a class was 41 (SD 20.02) students and the number of teachers per PE session was 2.25 (SD 0.77). Only 9 (25%) schools had specialist PE teachers teaching the PE lessons. In 21 (58.3%) of schools, class teachers taught PE, and in 6 (16.7) schools, it was both the class teacher and the specialist PE teacher (Table 3).

**Table 3:** Summary of the teacher and class related factors in the primary schools

Characteristics		Number (36)	Percentage (% = 100)
Type of PE Teacher who usually teaches PE	Class teacher	21	58.3
	PE Specialist	9	25.0
	Both	6	16.7
Number of Teachers available to teach PE in the school, Mean (SD)	5 (5.64)		
Teachers feel supported by the school administration to conduct PA	Yes	34	94.4
	No	2	5.6
Teachers attended further training courses in PE	Yes	29	80.6
	No	7	19.4
Number of times a year teachers attended further training courses in PE	None	7	19.4
	Once	17	47.2
	More than once	12	33.3
Number of PE teachers per session	1-2	26	72.2
	3-4	10	27.8
Schools with average size of class < 40 students	P.1	22	61.1
	P.2	28	77.8
	P.3	23	63.9
	P.4	22	61.1
	P.5	24	66.7
	P.6	26	72.2
	P.7	25	69.4

### School related factors

#### Time for physical education

PE was scheduled on the school timetable in 35 (97.2%) of the schools. In these schools it was compulsory, and they had a curriculum for PE. Most schools scheduled PE after lunch 16 (44%) and 5 (13.9%) schools had scheduled time for PE all through the day depending on the timetable.

Only 1 school had a full theory lesson of 40 minutes each week. All other 35 (97.2%) schools gave a five-minute theory session before the PE lessons.

PE was sometimes cancelled (mainly due to exams and rain) in all schools. In 1 school, there was more time for PE during exam time, because afternoons were free.

PE was reported on the end of term report cards of students in 13 (36.1%) of the schools mainly appearing only as a comment. Only 4 (11%) schools did not have a sports day.

### Availability of space for PA

Only 19 (52.8%) schools had space within the school in which the students could play and do PE. For those with no space, most went to a neighbouring field either by bus 4 (21%) or on foot 12 (63%) and 2 did the PE in class with the teachers improvising activities to get the children physically moving. 4 (11%) schools had a swimming pool, 2 schools had a tennis court and only 1 had an indoor gymnasium.

### Equipment and after-school activities for PA

Most schools 20 (55.6%) did not have enough equipment for PE, but since classes were on different days, they had some to use. Some improvised equipment 2 (6%). Repairs and maintenance were done on time in 26 (72.22%) of the schools usually dependent on the budget made at the beginning of each term. Only 28 (77.9%) of schools had optional after-school activities that were related to PA (See Table 4).

**Table 4:** Summary of the school-related factors

Characteristics	Level	Number (36)	Percentage (% = 100)
**Physical Education**			
**PE scheduled on the timetable**	Yes	35	97.2
	No	1	2.8
**Availability of Curriculum for PA**	Yes	35	97.2
	No	1	2.8
**The time when PE is scheduled**	Morning	16	44.4
	After a break or After lunch	5	13.9
	Any time	15	41.7
**What PE lessons involve**	Only theory lessons	0	0
	Only Physical Activity	11	30.6
	Both Theory and PA	25	69.4
**PE sometimes cancelled**	Yes	36	100.0
	No	0	0
**How often is PE cancelled**	Never	0	0
	Rarely	34	94.4
	Often	2	5.6
**Other school characteristics**			
**Availability of space on school grounds**	Yes	19	52.8
	No	17	47.8
**Availability of enough equipment for PA**	Yes	16	44.4
	No	20	55.6
**Availability of optional additional PA activities after school**	Yes	28	77.8
	No	8	22.2
**Availability of options for Special needs children**	Yes	13	36.1
	No	23	63.9
**Repairs and Maintenance were done on time**	Yes	25	71.4
	No	10	28.6
**The school has a sports day**	Yes	32	88.9
	No	4	11.1

### Factors associated with physical activity according to the WHO guidelines

Results from the bivariate level, unadjusted logistic regression indicated that school type, amount of school fees paid, school religion foundation, the time when PE is scheduled, what the PE lessons include, equipment availability, and availability of PE space be adjusted for provision of sufficient PA according to the WHO guidelines.

In the multivariate model, all factors were included. The time when PE was scheduled was statistically significantly related to adherence to WHO guidelines adherence (*p* < 0.05) by schools. Schools that reported that the PE was done after break or lunchtime were more likely to adhere to PE time guidelines compared to those that had PE time in the morning (OR = 19.2; *p*-value = 0.038) (Table 5).

**Table 5:** Unadjusted and adjusted models of factors predicting sufficient PA in Primary schools using WHO guidelines

	Unadjusted model				Adjusted model			
outcome	Odds Ratio	P > z	[95% Conf. Interval]		Odds Ratio	P > z	[95% Conf. Interval]	
**School type**								
Day	1.00	.	.	.	1.00	.	.	.
Mixed	1.00	1.000	0.21	4.81	0.08	0.273	0.00	6.99
**School fees**								
Less than 700,000	1.00	.	.	.	1.00	.	.	.
Greater or equal to 700,000	0.83	0.842	0.14	5.30	1.30	0.905	0.02	104.01
**PE time**								
Morning	1.00	.	.	.	1.00	.	.	.
After break or lunch	4.67	0.194	0.46	47.63	19.18	0.038*	1.50	25.00
All time	2.55	0.328	0.39	16.55	6.49	0.423	0.07	631.49
**PE coverage**								
Both	1.00	.	.	.	1.00	.	.	.
Only PA	0.70	0.700	0.12	4.19	8.22	0.323	0.13	537.98
**Volleyball**								
No	1.00	.	.	.	1.00	.	.	.
Yes	3.00	0.189	0.59	15.26	8.69	0.061	0.73	10.00
**Athletics**								
No	1.00	.	.	.	1.00	.	.	.
Yes	1.94	0.463	0.33	11.41	2.26	0.607	0.10	51.04
**PA space**								
No	1.00	.	.	.	1.00	.	.	.
Yes	1.67	0.534	0.33	8.35	13.27	0.137	0.44	39.70
**PE Teachers**	0.85	0.254	0.65	1.12	0.64	0.117	0.36	1.12
Student Teacher Ration	0.98	0.562	0.93	1.04	0.97	0.608	0.87	1.08
**_cons**					0.00	0.042	0.00	0.70

Important to note is that schools that had schools-fees greater than or equal to 700,000/= were more likely to adhere to WHO PE time guidelines (OR = 1.3; p-value = 0.905) compared to the schools paying less than 700,000/=, despite the relationship not being significant at (*p* > 0.05). The schools with athletics equipment were more likely to provide Sufficient PA according to the WHO guidelines (OR = 2.26; *p*-value = 0.607) compared to those without athletics equipment.

## Discussion

A major finding of this study was that the proportion of schools that provided sufficient time for PA according to the WHO guidelines before the pandemic was low (22.2%). Levels reported in this study are moderately similar to levels of adherence to guidelines reported in other countries including 5% in the USA, 27% in Australia, and 43% in Canada [23] all of which fall short of the recommended WHO guidelines. This is not surprising because of the challenges in implementing, monitoring, and adhering to country guidelines reported by UNESCO [7, 24].

The average scheduled time for PA for all schools was 197 minutes, well below the 300 minutes which is recommended by WHO; but surprisingly, well above the global average of 103 minutes weekly earlier reported [7]. These studies reflect that challenges to meet recommended guidelines and best practices need to be studied and understood in context and comprehensive school PA promotion interventions ought to be explored to support the increase of PA in primary schools [25, 26].

Although the literature shows that most schools aim to provide time for PA, irrespective of the time it appears on the timetable [27], a significant finding in this study was that schools that provided PE either after a break or after lunch were more likely to provide sufficient PE. Such a break likely allows schools to concentrate on schoolwork in the morning hours while allowing for sufficient engagement of students when their energy is waning. There is a need therefore to consider the exact time PE lessons are scheduled to ensure schools can provide sufficient time for PA. There is also a need to understand how time affects provision as well as if a specific time for PA yields better outcomes than another.

All schools provided a break time in which children had time to engage in active play after their snack. Although the time varied in schools, this was commendable because many schools in other countries do not have scheduled breaks for active play on the timetable [7, 27].

The schools offered activities as guided by the Uganda MoE&S which were divided into 3 categories: those involving balls, athletics, and gymnastics. All schools (100%) were able to offer at least one activity involving balls, much higher than a study done in neighbouring Kenya which showed that about 86% of schools had ball activities for children [28]. This performance may be attributed to ease of access to balls thus activities involving balls were more easily encouraged. In the regional context, 100% of primary schools in Asia, Europe, and N. America provided opportunities for such team sports, while in Africa, 97% of Primary schools offer such [7].

Although all schools provided football, interestingly, schools which also provided volleyball were 8 times more likely to meet the WHO guidelines for physical activity perhaps because volleyball has been reported to enhance the inclusion of more girls and hence more children in the PA activities [29]. This is critical because the ball activities are part of the organised sport which has been shown to have a high contribution towards overall PA in children [30]. Furthermore, football, volleyball, and other team sports are preferred activities by students and this preference has been shown to be sustained by adults in later years as well [31].

Schools which had provisions for athletics were twice as likely to provide sufficient time for PA according to the WHO guidelines. Although this factor was not statistically significant, this is an interesting find because ball games are known to provide more opportunities for PA compared to athletics and gymnastics [32].

This study also highlighted that in most schools (94.4%), the teachers felt supported by the School Administration to conduct PE. Perhaps one of the reasons was that 80.6% of schools recognised the importance of training and sent their PE teachers for further PE training each year with up to 33.3% of schools sending the teachers more than once each year. This is an encouraging find even though it was markedly different from other figures reported in the literature which indicate that in Africa and globally, there is a perceived low status accorded to PE activities and that teachers do not often feel supported [7, 33]. It should be noted that despite this support, the final model indicated no impact on PA suggesting that other factors may be more critical. Nonetheless, other researchers [34] report that in-service training is associated with a higher implementation of PA in schools; while [35, 36] recommended that a variety of interventions (including capacity building, offering choice and variety of activities, and embedding PE in curricular) ought to be considered by schools seeking to increase their PA.

In this study, PE was compulsory in 97.2% of schools, which is comparable to the global figure of 98.7% and slightly higher than what was overall reported in Africa (93%) [7]. All these schools followed the Uganda National Curriculum as required by the state and this is important because having a PA curriculum and ensuring PA inclusion on a timetable is a major recommendation to increase PA in schools [36].

In this study, 52.8% of schools had space within the schools for PE and active play which is in contrast to a US study where 99% of schools had access to a gym and 79% to school fields [37]. The odds of offering sufficient PA were greater in schools with a designated space for PA. Even though this was not statistically significant, studies have documented the availability of space as an important factor in enhancing PA in schools [23, 38], thus school administrators need to consider a broad range of factors in the implementation of PA in their contexts.

Schools which had 700,000/= or more for tuition and fees were more likely to provide sufficient PA which is in line with other studies [37, 39, 40] that have documented that a higher socioeconomic status may enhance the school’s ability to offer more PA due to availability of safe space, working equipment and more options which children could participate in. However, not all literature agrees that higher socioeconomic status is necessarily a determinant for schools providing PA opportunities [27].

The strength of the study was that it focused on the whole schools' ability to provide opportunity which had not yet been documented. The limitations of this study included recall bias likely affected participants’ responses since their answers depended on their ability to remember/recall events in the terms before the COVID pandemic and although the sample was representative, it was small and generated data may have been insufficient in providing a clearer picture.

## Conclusions

Only 22% of sampled schools offered sufficient time for PA in Primary schools in Kampala per WHO guidelines. Provision of sufficient time for PA was associated with PE lessons being offered either after break or lunch. However, sufficient PA was more likely in schools that had space for PA, schools which offered volleyball and athletics, and whose fees were 700,000/= ($183) or more.

## Data Availability

The dataset is readily available upon request from the lead author lmpalampa@yahoo.com

## Abbreviations

CDC: Centre for Disease Control
WHO: World Health Organisation
NCD: Non-communicable disease
PA: Physical Activity
PE: Physical Education
MOE&S: Ministry of Education and Sports
